# The broad spectrum of COVID-like patients initially negative at RT-PCR testing: a cohort study

**DOI:** 10.1186/s12889-021-12409-w

**Published:** 2022-01-07

**Authors:** Valeria Caramello, Alessandra Macciotta, Fabrizio Bar, Alessandro Mussa, Anna Maria De Leo, Alessandro Vincenzo De Salve, Fabio Nota, Carlotta Sacerdote, Fulvio Ricceri, Adriana Boccuzzi

**Affiliations:** 1 Emergency Department and High Dependency Unit, San Luigi Gonzaga University Hospital, Orbassano (TO), Italy; 2 Department of Clinical and Biological Science, University of Turin, Regione Gonzole 10, Orbassano (TO), Italy; 3 Unit of Cancer Epidemiology, Città della Salute e della Scienza University-Hospital, Turin, Italy; 4 Epidemiology Unit, Regional Health Service ASL TO3, Grugliasco (TO), Italy

**Keywords:** COVID-19, False-negative RT-PCR, Emergency department, Infection control, Isolation, Clinical judgment

## Abstract

**Background:**

Patients that arrive in the emergency department (ED) with COVID-19-like syndromes testing negative at the first RT-PCR represent a clinical challenge because of the lack of evidence about their management available in the literature.

Our first aim was to quantify the proportion of patients testing negative at the first RT-PCR performed in our Emergency Department (ED) that were confirmed as having COVID-19 at the end of hospitalization by clinical judgment or by any subsequent microbiological testing. Secondly, we wanted to identify which variables that were available in the first assessment (ED variables) would have been useful in predicting patients, who at the end of the hospital stay were confirmed as having COVID-19 (false-negative at the first RT-PCR).

**Methods:**

We retrospectively collected data of 115 negative patients from2020, March 1st to 2020, May 15th. Three experts revised patients’ charts collecting information on the whole hospital stay and defining patients as COVID-19 or NOT-COVID-19. We compared ED variables in the two groups by univariate analysis and logistic regression.

**Results:**

We classified 66 patients as COVID-19 and identified the other 49 as having a differential diagnosis (NOT-COVID), with a concordance between the three experts of 0.77 (95% confidence interval (95%CI) 0.66- 0.73). Only 15% of patients tested positive to a subsequent RT-PCR test, accounting for 25% of the clinically suspected. Having fever (odds ratio (OR) 3.32, (95%CI 0.97-12.31), *p* = 0.06), showing a typical pattern at the first lung ultrasound (OR 6.09, (95%CI 0.87-54.65), *p* = 0.08) or computed tomography scan (OR 4.18, (95%CI 1.11-17.86), *p* = 0.04) were associated with a higher probability of having COVID-19.

**Conclusions:**

In patients admitted to ED with COVID-19 symptoms and negative RT-PCR a comprehensive clinical evaluation integrated with lung ultrasound and computed tomography could help to detect COVID-19 patients with a false negative RT-PCR result.

**Supplementary Information:**

The online version contains supplementary material available at 10.1186/s12889-021-12409-w.

## Background

Severe acute respiratory syndrome coronavirus 2 (SARS-CoV-2) and the related Coronavirus Disease 19 (COVID-19) emerged as a global pandemic in March 2020. Piedmont was the second most affected area in Italy, which was one of the most impacted among European countries in the earliest phases. Pandemic management emphasized accurate case identification and containment; the risk of in-hospital outbreaks was a great concern. A reliable case identification at hospital admission is crucial to provide the best quality of care, to reduce nosocomial infections and the risk of contagion to hospital staff. A foremost priority of Emergency Departments (EDs) was first the rapid detection of COVID-19 patients in order to keep them separate for the entire duration of their time in the ED and later to select an appropriate admission ward.

SARS-CoV-2 RNA detection by Reverse Transcriptase Polymerase Chain Reaction (RT-PCR) from an upper airways (nasal and/or throat) swab is the gold standard for case confirmation which is used to rule out infections among high-risk populations (symptomatic patients, those exposed to confirmed cases and health care workers) [1–4]. RT-PCR has shown high sensitivity and specificity [1, 5] however its diagnostic accuracy may be lower than optimal [6, 7]. A good technique for collection, handling, identification, transport, storage and analysis [8] of the swabs in accordance with WHO recommendations [9] could impact on the pre-analytical and analytical problems that have been shown to reduce RT-PCR reliability [8, 10–13].

All these technical issues could have been worse in the first phase of the pandemic because laboratories worked under high workloads and were overwhelmed by the high number of cases [8]. However, the situation improved as the regional health system increased the number of microbiology laboratories that could perform RT-PCR. False-negatives occurred in 2 to 29% of cases in the Chinese studies from the first period of the pandemic analysed by Arevalo Rodriguez in a recent meta-analysis [14]. The false-negative prevalence may be further underestimated because most studies defined COVID cases with at least one positive RT-PCR, meaning that patients who never tested positive would not be included [2].

The infectivity of these false-negative patients, although previously described by case reports [15] is still controversial. In a recent review, Kucirca et al. suggested that high-risk patients should be considered as false-negatives and submitted to further testing, because RT-PCR accuracy changes with the pre-test probability of infection [2]. Moreover, they showed that RT-PCR results are strictly related with time since exposure or symptom onset. The timing of the test also needs to be considered as well as the window of viral replication in order to guide decisions regarding isolation discontinuation [2–16].

After Xie et al. first described the case of five “false negative” patients [6] detected by chest computed tomography (CT), many other similar reports have been published [7, 17–20]. Areas of ground-glass opacity (GGO) or a “crazy paving” pattern at chest CT [21] became the alternative gold-standard, or even the inclusion criteria as in the study by Baicry et al. [17]. Although the American College of Radiology reiterated the warning that a CT scan should not be used as a first-line test, their guidelines [22] specifically provided guidance to radiologists reporting CT findings that were potentially attributable to COVID-19 pneumonia and this classification has been widely used [7, 17–19, 21–23].

Many authors suggest that patients with COVID-19-like symptoms and imaging should undergo repeated RT-PCR testing [14, 15, 19, 21] whereas others highlight the utility of detecting the virus in lower respiratory tract specimens [24, 25] or in different tissues to detect viral shedding [26]. Early antibody testing has been suggested in patients with clinically suspected COVID-19 with repeated negative swabs [27, 28].

In the earliest phases of the pandemic in Italy, antibody testing, antigenic testing and other point-of-care diagnostic methods were not available. Moreover, the accuracy of these rapid tests is still controversial, whereas the most efficient methods for antibody testing and RT-PCR itself could take hours before a result is obtained [29]. The WHO advises against the use of serology alone to diagnose COVID-19 and suggests that results should be interpreted by taking into account several factors including the timing of the disease, clinical morbidity, epidemiology and prevalence within the setting, type of test used, validation method and reliability of the results [29, 30]. The duration of the persistence of antibodies generated in response to SARS-CoV-2 infection and their effectiveness in offering protective immunity is still under study [31, 32]. In our experience in Piedmont, it was not uncommon to have COVID-19-like patients that repeatedly tested negative at RT-PCR, as observed by other authors [17, 33, 34].

Due to the great uncertainty at the very beginning of the epidemic, we faced both a clinical diagnostic dilemma (−Is this COVID-19-like syndrome with negative RT-PCR a “false negative”?-) and a bigger organizational dilemma (−Where should we admit a suspected COVID-19 with a negative swab?-). Infact, in patients negative to the first RT-PCR, further testing and second-level examinations could have led to a differential diagnosis or have confirmed COVID-19, however this process could sometimes take the whole hospitalization period. The duty of the Emergency Department is also to prevent SARS-CoV2 infection in a possibly negative patient and at the same time, in other patients sharing the same area during the hospital stay. In our institution, we created a special ward with single-room occupancy, however we struggled to correctly select patients for this limited resource.

We decided to analyze the data of patients presenting to the ED, during the first pandemic wave, with COVID-19-like syndromes that tested negative at the first RT-PCR, in order to:

1. quantify the proportion of patients that were confirmed COVID-19 by any subsequent RT-PCR testing (on nasal and/or lower airway specimens);
2. quantify the proportion of patients with only clinical confirmation of COVID-19 at the end of the hospital stay;
3. identify if any variables available in the first ED assessment of these patients could have been associated with a higher probability of COVID-19 confirmation at the end of the hospital stay.

## Methods

We retrospectively searched the ED database for patients that accessed the ED of the San Luigi Gonzaga Hospital in Orbassano (TO), northern of Italy, during the first wave of the COVID-19 pandemic (1 March - 15 May 2020) and that tested negative to RT-PCR for SARS-CoV2, but were diagnosed as suspected COVID-19 and admitted to an isolation room due to the negative swab. Seven more patients with mild COVID-19-like syndromes that had been discharged in preventive home-isolation were also included. All patients signed a consent form at arrival and for the disclosure of personal information for public health purposes and research. The study was approved by the Institutional Ethics Committee (communication 13/2020, n°11,435, 03/09/2020).

Eligible patient charts were reviewed by three independent researchers and were included in the study if the following criteria were met:

- Epidemiological criteria (exposure to COVID-19 cases, nursing-home resident, health workers)
- Clinical criteria at admission (symptoms such as fever, cough, dyspnoea, respiratory failure, loss of taste or smell)

We defined the study design, variables, objectives and trained four data collectors with experience in research in emergency medicine. Each patient’s ED record was examined by two different researchers and the data collected using a chart review form (CRF). If any disagreement occurred regarding chart interpretation, a third more experienced researcher reconsidered the chart. The reliability and performance of data collection were regularly monitored by supervisors.

For all eligible patients we recorded the data measured in the first hours in the ED (ED variables): demographic data, comorbid conditions, symptoms at presentation, time from symptom onset, results of laboratory testing, results of imaging (bedside lung ultrasound, chest X-ray and CT scan if available) and the result of the first RT-PCR.

All ED staff were trained in the technique to perform swab collection according to the subsequent updates of the WHO recommendations to ensure the highest accuracy. We collected nasopharyngeal and oropharyngeal swabs at the beginning of March and then only nasopharyngeal swabs [31, 35]. Samples were immediately sent to the microbiology laboratory following recommendations to reduce pre-analytical problems. RT-PCR was performed according to Corman et al. following WHO recommendations [1, 35].

We collected the results of the first and any subsequent RT-PCR test and all the other microbiological tests for differential diagnosis of pneumonia (such as cultures and tests for other respiratory pathogens, RT-PCR for SARS CoV-2 on bronchoalveolar lavage/lower airway specimens, blood cultures, search for Legionella and Pneumococcal antigens). We also collected information regarding the oxygen support provided, together with the need for continue positive airway pressure (CPAP) support, non-invasive mechanical ventilation (NIMV) and invasive mechanical ventilation (IMV) and relative duration. Finally, we registered information on admission, hospitalization in a general ward or high intensity of care ward and its duration, length of hospital stay, in-hospital mortality and discharge at home or to a rehabilitation facility.

To define the clinical confirmation of COVID-19 we searched records of the entire hospital stay for any clinical, laboratory and imaging data that could confirm a COVID-19 diagnosis or suggest a differential diagnosis.

A second group of three experienced researchers, aware of the relevant literature, but blinded to the study objective, independently evaluated the discharge summary and all the hospital records of each patient. Then they expressed a judgment of the likelihood of COVID-19 (COVID) or a different diagnosis from COVID-19 (NOT-COVID). We considered a patient to be COVID by clinical judgment when at least two out of three experts agreed on the definition. If at least two out of three experts agreed on a differential diagnosis, we considered the patient as NOT-COVID.

Data were collected, held and analyzed anonymously. Data were described as medians and interquartile ranges for quantitative variables and as absolute frequencies and percentages for categorical variables. We performed univariate comparisons for the variables available in the ED (ED variables) in the group of COVID versus NOT-COVID. Based on the non-normal distribution of the data assessed by the Kolmogorov-Smirnov test, univariate comparisons were performed using the Wilcoxon sum rank test. For categorical variables, we used the chi-square test or Fisher exact test when the hypotheses for conducting a chi-square test were not met. The concordance in clinical judgment was tested using Fleiss K and the 95% confidence interval..

Lately, a logistic regression was performed and odds Ratios (ORs) and 95% confidence intervals (95% CI) were computed, using the ED variables that were significantly different in the two groups. The aim was to determine the probability ratio of having COVID-19 and the relative weight of each variable.

Finally, we run a sensitivity analysis in patients with microbiological confirmed COVID-19 (by a positive RT-PCR test, or positive BAL test or antibodies presence on serological testing) to reduce possible inclusion biases.

All tests were two-sided and a *p*-value of 0.05 was considered significant. Analyses were performed using R version 3.4.2.

## Results

From 2020, March 1st to 2020, May 15th we admitted 108 patients to isolation rooms, who tested negative at the first RT-PCR, 7 further negative patients were discharged in isolation at home with a diagnosis of suspected COVID-19.

All patients showed at least one criterion for inclusion: 50 (43%) patients had an epidemiological criterion, 106 (92%) patients had a clinical criterion, and 41 (35%) had both. Table 1 describes the prevalence of each of the inclusion criteria in our cohort. A higher proportion of COVID-19 patients reported a fever, whereas other complaints were similarly distributed among the COVID-19 and NOT-COVID groups.

**Table 1:** Inclusion criteria of RT-PCR negative patients. Data are described in the whole group and in the two subgroups COVID-19 and NOT-COVID defined at the end of the hospital stay by clinical judgment or further testing. Data are expressed as absolute frequencies and percentages (in brackets) for categorical variables and as medians and Interquartile Ranges [IQR]

	Overall *Median [IQR] N (%)*	COVID-19 *Median [IQR] N (%)*	COVID-free/other than COVID *Median [IQR] N (%)*	***p***
**n**	115	66	49	
**Epidemiological Criteria**				0.074°
*No*	65 (56.5)	42 (63.6)	23 (46.9)	
*Yes*	50 (43.5)	24 (36.4)	26 (53.1)	
**Epidemiological Criteria Specification**				0.135^§^
*Contact to COVID-19*	14 (12.2)	8 (12.1)	6 (12.2)	
*Nursing home resident*	19 (16.5)	8 (12.1)	11 (22.4)	
*Repeated health care services users* *(dialysis, day hospital)*	12 (10.4)	4 (6.1)	8 (16.3)	
*Health worker*	5 (4.3)	4 (6.1)	1 (2.0)	
*None*	65 (56.5)	42 (63.6)	23 (46.9)	
**Clinical criteria**				0.005^§^
*No*	9 (7.8)	1 (1.5)	8 (16.3)	
*Yes*	106 (92.2)	65 (98.5)	41 (83.7)	
***Clinical criteria specification***				
**Cough, Dyspnoea**				0.242°
*No*	40 (34.8)	20 (30.3)	20 (40.8)	
*Yes*	75 (65.2)	46 (69.7)	29 (59.2)	
**Fever**				0.001°
*No*	53 (46.1)	22 (33.3)	31 (63.3)	
*Yes*	62 (53.9)	44 (66.7)	18 (36.7)	
**Hyposmia, Hypogeusia**				0.392^§^
*No*	110 (95.7)	62 (93.9)	48 (98.0)	
*Yes*	5 (4.3)	4 (6.1)	1 (2.0)	
**Nausea, Vomiting, Diarrhoea**				1.000^§^
*No*	108 (93.9)	62 (93.9)	46 (93.9)	
*Yes*	7 (6.1)	4 (6.1)	3 (6.1)	
**Respiratory failure**				0.281°
*No*	55 (47.8)	29 (43.9)	26 (54.2)	
*Yes*	59 (51.3)	37 (56.1)	22 (45.8)	
**Number of symptoms**	2.00 [1.00 - 2.00]	2.00 [1.00 - 3.00]	2.00 [1.00 - 2.00]	0.003^#^
**Worsening of PO2/FiO2 without cause**				1.000°
*No*	63 (54.8)	36 (64.3)	27 (64.3)	
*Yes*	35 (30.4)	20 (35.7)	15 (35.7)	

Comparisons are made with: ^#^Wilcoxon Rank Sum Test; ^°^Chi-Square test; ^§^Fisher’s Exact test

The hospital records and the discharge summary were reviewed for all 115 patients. The three experienced physicians showed a good concordance in diagnosing COVID-19 (k 0.767 (95%CI 0.662- 0.873). A total of 51 patients were clinically considered to be COVID-19 by all three evaluators, whereas 44 patients were considered NOT-COVID by all three evaluators; 11 patients were considered COVID-19 by two out of three evaluators, and were thus included in the COVID-19 group, whereas nine patients were judged COVID-19 by only one expert and were thus included in the NOT-COVID group.

One hundred and eight patients out of 115 patients underwent a second swab after a median of 2 [1 - 4] days, 72 patients underwent a third swab after a median of 3 [2 - 9] days, and 30 underwent a fourth swab after a median of 6 [2 - 9] days.

**Fig. 1:**
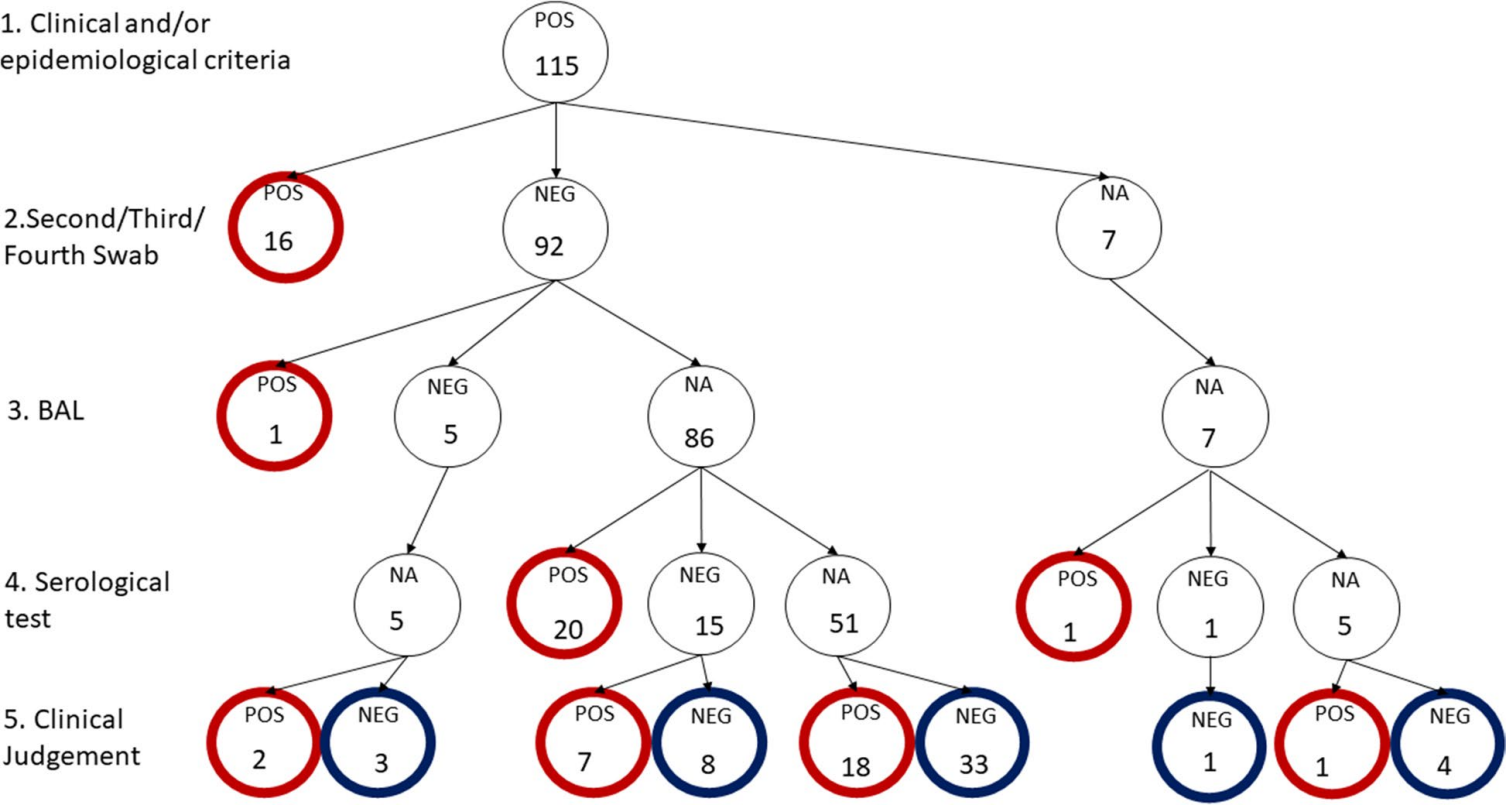
Further testing of patients and their results in a decision tree in order to define the two groups as COVID-19 vs NOT-COVID after the entire hospital stay

Overall 38/115 (33%) patients were positive at further testing performed during hospitalization: one tested positive at BAL, 14 tested positive at a subsequent RT-PCR swab test, two tested positive at a subsequent RT-PCR and then showed antibodies in a further assay, 20 had a repeatedly negative RT-PCR and showed the presence of antibodies, and one patient did not repeat RT-PCR but showed the presence of antibodies. Figure 1 presents the further testing of patients and their results in a decision tree in order to define the two groups as COVID-19 vs NOT-COVID after the entire hospital stay. Due to the retrospective nature of the study seven patients did not undergo a further swab and 61 patients were not tested for the presence of antibodies. In summary, 66 (57%) patients were finally considered as presenting COVID-19 (38/66 by further testing and 28/66 by clinical judgment only); the remaining 49 patients (43%) who at arrival were suspected of COVID-19, were finally diagnosed to have a disease other than COVID-19 (NOT-COVID group). The clinical judgment was accurate in identifying 34 out of 38 patients who were found to be positive to further testing. In the subset of 77 patients who never tested positive for SARS-CoV-2 either by RT-PCR or by antibodies (because they were negative or the results were unavailable) the agreement among clinicians was even higher (K = 0.84, 95%CI (0.72-0.96)).

We compared the ED variables collected in the two groups described above (COVID-19 vs NOT-COVID) in order to identify those that could help diagnose COVID-19 in the first few hours after hospital arrival.

The COVID-19 group was significantly younger than the NOT-COVID group (72.76 [55 - 81] vs 78.09 [72 - 84], *p* = 0.022) and a slight but non-significant male prevalence was observed in the COVID-19 group. COPD showed a higher prevalence in the NOT-COVID group (16 (32.7%) vs 6 (9.1%), *p* = 0.001), whereas no significant differences were observed for any other pre-existing comorbid conditions. COVID-19 patients showed a lower value of total white blood cell count (median 7.62 [5.12 - 11.50] × 10^3^/mcl vs 10.90 [7.48 - 15.20] × 10^3^/mcl, p 0.005). Details could be found in Table 2. On the other hand, no significant differences were observed in inflammatory markers, lactate dehydrogenase levels nor in other laboratory tests at arrival (details in Table 3). Similarly, both groups had comparable PaO2/FiO2 ratios at the first arterial blood gas analysis at arrival.

**Table 2:** Demographic, comorbid conditions and first laboratory tests in ED of RT-PCR negative patients. Data are described in the whole group and in the two subgroups COVID-19 and NOT-COVID defined at the end of the hospital stay by clinical judgment or further testing. Data are expressed as absolute frequencies and percentages (in brackets) for categorical variables and as medians and Interquartile Ranges [IQR]

	Overall *Median [IQR] N (%)*	COVID-19 *Median [IQR] N (%)*	COVID-free/other than COVID *Median [IQR] N (%)*	***p***
**n**	115	66	49	
**age (years)**	76.17 [58 - 84]	72.76 [55- 81]	78.09 [72 - 84]	0.022^#^
**sex**				0.275°
*F*	45 (39.1)	23 (34.8)	22 (44.9)	
*M*	70 (60.9)	43 (65.2)	27 (55.1)	
**Hypertension**				0.997°
*No*	54 (47.0)	31 (47.0)	23 (46.9)	
*Yes*	61 (53.0)	35 (53.0)	26 (53.1)	
**Cardiopathy**				0.342°
*No*	79 (68.7)	43 (65.2)	36 (73.5)	
*Yes*	36 (31.3)	23 (34.8)	13 (26.5)	
**COPD**				0.001°
*No*	93 (80.9)	60 (90.9)	33 (67.3)	
*Yes*	22 (19.1)	6 (9.1)	16 (32.7)	
**Lung fibrosis**				0.162^§^
*No*	110 (95.7)	65 (98.5)	45 (91.8)	
*Yes*	5 (4.3)	1 (1.5)	4 (8.2)	
**Lung Cancer**				1.000^§^
*No*	108 (93.9)	62 (93.9)	46 (93.9)	
*Yes*	7 (6.1)	4 (6.1)	3 (6.1)	
**Cancer**				0.795°
*No*	95 (82.6)	54 (81.8)	41 (83.7)	
*Yes*	20 (17.4)	12 (18.2)	8 (16.3)	
**Immunodepression**				0.210°
*No*	95 (82.6)	52 (78.8)	43 (87.8)	
*Yes*	20 (17.4)	14 (21.2)	6 (12.2)	
**Neurological disorders**				0.192°
*No*	78 (67.8)	48 (72.7)	30 (61.2)	
*Yes*	37 (32.2)	18 (27.3)	19 (38.8)	
**Diabetes or other metabolic conditions**				0.627°
*No*	71 (61.7)	42 (63.6)	29 (59.2)	
*Yes*	44 (38.3)	24 (36.4)	20 (40.8)	
**Renal Failure**				0.144°
*No*	100 (87.0)	60 (90.9)	40 (81.6)	
*Yes*	15 (13.0)	6 (9.1)	9 (18.4)	
**Total WBC count /μl**	9.24 [6.02 - 13.38]	7.62 [5.12 - 11.50]	10.90 [7.48 - 15.20]	0.005^#^
**Lymphocyte count/ μl**	1175.00 [792.50 - 1765.00]	1120.00 [820.00 - 1710.00]	1410.00 [740.00 - 1870.00]	0.567^#^
**LDH** U/L	285.50 [228.00 - 393.00]	288.50 [243.75 - 380.75]	272.00 [217.25 - 427.50]	0.351^#^
**PCR** mg/dL	4.46 [1.02 - 13.16]	5.64 [2.15 - 13.23]	3.48 [0.49 - 12.60]	0.257^#^
**PCT** ng/mL	0.07 [0.03 - 0.55]	0.05 [0.04 - 0.26]	0.16 [0.03 - 1.05]	0.151^#^
**PaO2/FiO2 at arrival**	318 [242 - 357]	331 [252 - 385]	322 [247 - 373]	0.350^#^

Comparisons are made with: ^#^Wilcoxon Rank Sum Test; ^°^Chi-Square test; ^§^Fisher’s Exact test

**Table 3:** Imaging at arrival in the ED of RT-PCR negative patients. Data are described in the whole group and in the two subgroups COVID-19 and NOT-COVID defined at the end of the hospital stay by clinical judgment or further testing. Data are expressed as absolute frequencies and percentages (in brackets) for categorical variables and as medians and Interquartile Ranges [IQR]

	Overall *N (%)*	COVID-19 *N (%)*	COVID-free/other than COVID *N (%)*	***p***
**Chest X ray (*n*** **= 107)**				0.525°
*Pneumonia consolidation*	24 (22.4)	15 (24.6)	9 (19.6)	
*Interstitial syndrome*	29 (27.1)	19 (31.1)	10 (21.7)	
*Aspecific findings*	30 (28.0)	15 (24.6)	15 (32.6)	
*Normal CXR*	24 (22.4)	12 (19.7)	12 (26.1)	
**Lung ultrasound (*n*** **= 88)**				0.019°
*Consolidation*	18 (20.5)	13 (23.6)	5 (15.2)	
*Monolateral mild interstitial syndrome*	11 (12.5)	5 (9.1)	6 (18.2)	
*Bilateral severe interstitial syndrome*	33 (37.5)	26 (47.3)	7 (21.2)	
*Pleural effusion*	13 (14.8)	7 (12.7)	6 (18.2)	
*Normal Lung Ultrasound*	13 (14.8)	4 (7.3)	9 (27.3)	
**CT scan (*n*** **= 85)**				< 0.001^§^
*Typical pattern*	37 (43.5)	29 (58.0)	8 (22.9)	
*Atypical pattern*	19 (22.4)	5 (10.0)	14 (40.0)	
*Undetermined*	27 (31.8)	16 (32.0)	11 (31.4)	
*Normal CT scan*	2 (2.4)	0 (0.0)	2 (5.7)	
**Irregular pleural line (*n*** **= 89)**				0.043^§^
*No*	82 (92.1)	49 (87.5)	33 (100.0)	
*Yes*	7 (7.9)	7 (12.5)	0 (0.0)	

Comparisons are made with: ^°^Chi-Square test; ^§^Fisher’s Exact test

With regards to imaging results, the presence of severe interstitial syndrome at the first lung ultrasound was significantly higher in COVID-19 patients, followed by consolidation. An irregular pleural line at ultrasound was present only in COVID-19 patients. The first computed tomography scan showed a typical pattern in the majority of COVID-19 patients whereas an atypical or undetermined pattern was shown in NOT-COVID-patients. (details in Table 3). Due to the retrospective nature of the study, a chest x-ray was performed in 107/115 patients, a lung ultrasound in 88/115 and a CT scan in 85/115.

Concerning the clinical course, COVID-19 patients showed no significant differences in the need for CPAP, nor duration of CPAP support. No differences were found in high dependency unit occupancy or length of stay in intensive care wards (details in Table 4). In the whole group, we observed a mortality rate of 21% (24 patients died during hospitalization): COVID-19 patients had a higher mortality rate (18 (29%) vs 6 (13%) and fewer were discharged home after the hospital stay (44 (71%) vs 40 (87%); *p* = 0.08).

**Table 4:** Clinical course of RT-PCR negative patients. Data are described in the whole group and in the two subgroups COVID-19 and NOT-COVID defined at the end of the hospital stay by clinical judgment or further testing. Data are expressed as absolute frequencies and percentages (in brackets) for categorical variables and as medians and Interquartile Ranges [IQR]

	Overall *Median [IQR] N (%)*	COVID-19 *Median [IQR] N (%)*	COVID-free/other than COVID *Median [IQR] N (%)*	***p***
**CPAP/NIV**				0.961°
*No*	96 (83.5)	55 (83.3)	41 (83.7)	
*Yes*	19 (16.5)	11 (16.7)	8 (16.3)	
**CPAP/NIV duration days**	5.00 [2-8]	4.00 [2-7]	5.50 [3-8]	0.803^#^
**ED outcome**				1.000^§^
*Discharged*	7 (6.1)	4 (6.1)	3 (6.1)	
*Admitted*	108 (93.9)	62 (93.9)	46 (93.9)	
***Hospital admission and outcome***				
**Hospital ward**				0.169^§^
*IOT/ICU transfer*	4 (3.7)	2 (3.2)	2 (4.3)	
*HDU*	22 (20.4)	9 (14.5)	13 (28.3)	
*Regular Ward*	82 (75.9)	51 (82.3)	31 (67.4)	
**Length of stay in HDU**	7.50 [4 - 21]	7.50 [4 - 21]	7.50 [4 - 20]	0.702^#^
**Hospital outcome**				0.081^°^
*discharged*	84 (77.8)	44 (71.0)	40 (87.0)	
*dead*	24 (22.2)	18 (29.0)	6 (13.0)	

Comparisons are made with: ^#^Wilcoxon Rank Sum Test; ^°^Chi-Square test; ^§^Fisher’s Exact test

The sensitivity analysis in patients with microbiological confirmed COVID-19 compared with NOT-COVID group, confirmed the previous findings (Supplementary Table 1).

**Fig. 2:**
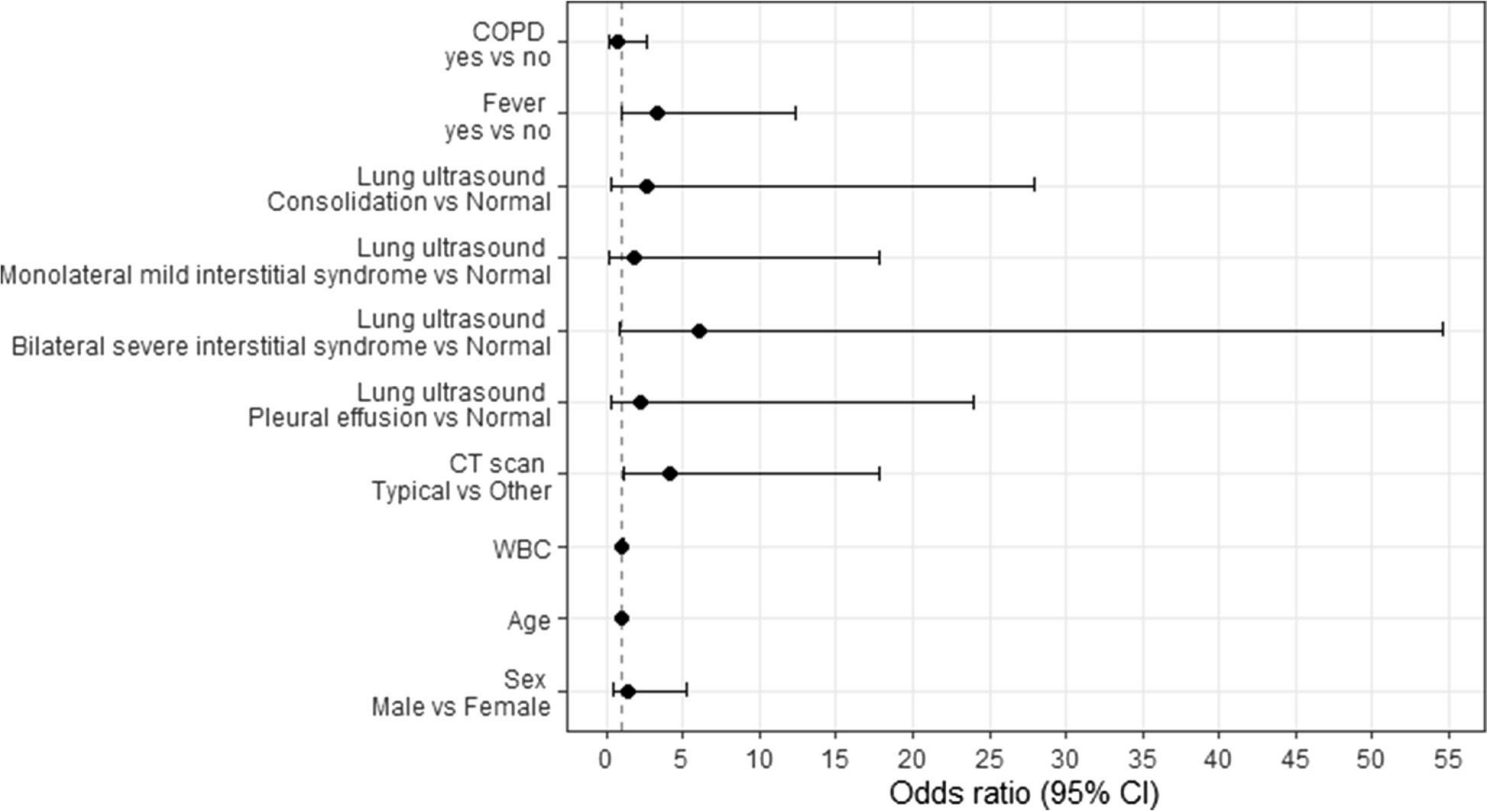
Visual representation of the ORs and 95% CI of the logistic regression presented in Table 5

The results of the logistic regression to investigate the association between ED variables and COVID-19 patients are shown in Fig. 2 and Table 5. Patients who reported a fever had 3.32 [95% CI 0.97-12.31] times the risk of having COVID-19. A bilateral severe interstitial syndrome at lung ultrasound showed the highest OR of having COVID-19 (6.09 [95% CI 0.87-54.65]); a typical pattern at CT had 4.18 [95% CI 1.11-17.86] times the risk of having COVID-19. Likewise, the sensitivity analysis showed similar results (Supplementary Table 2).

**Table 5:** Logistic regression of ED variables for the diagnosis of COVID. Odds ratio and confidence interval (CI at 95%) are shown and relative *p* values

		OR	95% confidence interval	***p*** value
**COPD**	*yes* vs *no*	0.66	(0.16-2.69)	0.561
**fever**	*yes* vs *no*	3.32	(0.97-12.31)	0.061
**Lung ultrasound**	*Consolidation* vs *Normal Lung Ultrasound*	2.58	(0.27-28)	0.414
	*Monolateral mild interstitial syndrome* vs *Normal Lung Ultrasound*	1.81	(0.21-17.88)	0.596
	*Bilateral severe interstitial syndrome* vs *Normal Lung Ultrasound*	6.09	(0.87-54.65)	0.080
	*Pleural effusion* vs *Normal Lung Ultrasound*	2.27	(0.26-23.92)	0.470
**CT scan**	*Typical* vs *Other*	4.18	(1.11-17.86)	0.040
**WBC**		1.01	(0.98-1.06)	0.651
**age**		0.99	(0.95-1.03)	0.617
**sex**	*Male* vs *Female*	1.45	(0.41-5.19)	0.557

## Discussion

The diagnostic confirmation of patients who arrived in the ED with COVID-19-like syndromes but tested negative at the first RT-PCR is a challenge for the emergency physician due to the consequences for individual patient care, infection control, scarce resources (hospital isolation rooms) and public health measures [3, 14–17]. Further examinations and repeated testing during hospitalization could help in the diagnosis, however, in the meantime, it is essential to maintain a high index of suspicion and a separate pathway for falsely negative cases [19]. We evaluated a cohort of RT-PCR negative patients the majority of whom presented in the ED with symptoms consistent with COVID-19-like syndrome or had known epidemiological factors. These two inclusion criteria defined a high-risk cohort that was managed throughout the hospital stay in an area dedicated to a single room occupancy.

There is a lack of evidence regarding the appropriate treatment of COVID-19-like patients in the absence of microbiological confirmation, together with the appropriate isolation measures and duration; furthermore, those patients are excluded from trials [2, 14]

e observed a very high ratio of patients that persistently tested negative at a further swab, but had a clinical illness that mimicked COVID-19 and in whom we ruled-out other differential diagnoses. In fact in our cohort, less than 15% of patients were positive at a further RT-PCR test on a nasal or lower airway specimen, accounting for only 25% of those clinically suspected. More than half of the remaining uncertain cases (98 patients) were clinically confirmed to be COVID-19, after further examination at the end of the hospital stay.

In many cases, diagnosis relies only on clinical judgment, which fortunately showed a good concordance among the three evaluators and agreed with the further testing in most cases.

Our findings were recently confirmed by other authors who found a 19-27% of patients with a negative RT-PCR but similar clinical and biochemical characteristics and a similar prognosis of COVID-19 illness [36, 37]; others derived from the clinical characteristics they observed a clinical rule to predict risk of COVID-19 [38].

We suggest maintaining a higher suspicion in patients with COVID-19-like syndromes who test negative at the first RT-PCR and that clinical findings together with microbiological results should guide isolation precautions.

We confirmed that imaging tests could be useful for COVID-19 identification. Typical CT scan patterns and the presence of a bilateral interstitial syndrome at lung ultrasound seemed to be associated with a higher probability of having COVID-19. We suggest the use of a bedside point-of-care ultrasound (LUS) during the first ED examination, searching for signs of interstitial pneumonia or a differential diagnosis of dyspnoea, in agreement with Pivetta et al. [39]. LUS can be performed rapidly, integrated with a clinical evaluation and with a careful epidemiological and clinical history, and thus provide rapidly available information on the risk of having COVID-19, before and regardless of the RT-PCR results. The “light-beam” sign corresponds to the early appearance of “ground-glass” alterations at CT scan, which was found to be very specific for a COVID-19 diagnosis [40–42]. A CT scan performed at arrival is useful in patients with negative RT-PCR in order to identify pneumonia that could be associated with COVID-19. The classification in patterns that we adopted according to the American College of Radiology guidelines was also effective in our cohort [22].

Baicry et al. [17] used typical CT patterns as inclusion criteria to select their cohort of false-negative COVID-19 patients and CT is frequently used as an alternative “gold-standard” [7, 17–23]. Nevertheless, CT should not be used as a first-line test to diagnose COVID-19, because CT findings are not specific and are useful for the “rule in” of COVID − 19 only if the pre-test probability of COVID-19 is high, as in the worst phases of the pandemic [43, 44].

We found that in our cohort clinical judgement as a surrogate gold-standard was useful to include cases with a mild extension of pneumonia. On the other hand, if only patients with a typical CT scan are considered, more severe cases could be selected. To the best of our knowledge, this is the first study to include patients with a negative RT-PCR with different illness severity and clinical presentation.

COVID-19 presents with non-specific symptoms and a broad spectrum of severity. Neither the symptoms nor the severity are predictive of a positive RT-PCR or of a false-negative result [45]. This represents a major limitation for emergency physicians and was also confirmed in our cohort, with the possible exception of fever. Laboratory tests and biomarkers have been shown to be altered in the most severe COVID-19 cases and are useful for prognostic purposes but are not pathognomonic for COVID-19 [17, 46, 47]. Laboratory tests and respiratory parameters in our cohort of suspected COVID-19 proved to be poor in predicting the diagnosis, probably because of the great heterogeneity in individual values and the mixed severity of cases in our cohort. In both COVID-19 and NOT-COVID groups, we found that most of the patients needed hospital admission, in 20% of cases in High Dependency Unit, whereas, similarly to other authors [36, 37], we identified a trend toward higher mortality in COVID-19 patients. Recently, Alfadda et al. found that an advanced age and having many comorbid conditions was associated with having a false negative RT-PCR [36]. Interestingly, in our cohort, COPD was the only chronic respiratory condition that prevailed in NOT-COVID patients, which in contrast showed no significantly higher prevalence of cardiopathy, lung cancer or lung fibrosis.

### Strengths

We believe that our work is original because we have tried to make up for the lack of evidence on patients who repeatedly tested negative to RT-PCR and that could manifest with different COVID-19 phenotypes. In such cases, the diagnostic definition may need an integrated approach that takes time and resources. The strength of our study lies in the accurate case evaluation by three experienced physicians who independently reviewed the clinical charts. The good concordance among the experts led to an accurate group definition by clinical judgment.

### Limitations

The main limitation of the study is that in this cohort of COVID-like patients with a negative RT-PCR, a “gold-standard” for the confirmation of COVID-19 was lacking. Although a clinical judgment is less objective, our included all the information obtained throughout the hospital stay. The sensitivity analysis was useful to confirm the validity of this approach. Another limitation was the rate of missing data due to the retrospective nature of the study. In fact nearly 30% of patients did not undergo a lung ultrasound or a CT scan in the ED, which limited statistical significance. In terms of a diagnostic definition, seven (6%) patients did not undergo a further swab test and 61 (53%) patients were never tested for antibodies. In hindsight, we would have performed more antibody testing if it had been available, but it was approved only later.

## Conclusion

We tried to evaluate which clinical variables that were readily available in the first hours of the hospital stay, were associated with having COVID-19 in this cohort of patients who were initially negative at RT-PCR testing. A clinical prediction rule combining clinical findings with lung ultrasound and CT scan in the ED could be used to define safe and appropriate patient pathways and location especially when health care systems are overwhelmed and sophisticated testing is not available or takes a long time.

## Supplementary Information

**Additional file 1: Table S1.** Sensitivity analyses. Data are described in the two subgroups COVID-19 (excluding those defined only by clinical judgement) and NOT-COVID defined at the end of the hospital stay by clinical judgment or further testing. Data are expressed as absolute frequencies and percentages (in brackets) for categorical variables and as medians and Interquartile Ranges [IQR]. Comparisons are made with: #: Wilcoxon Rank Sum Test; °: Chi-Square test; §: Fisher’s Exact test. **Table S2.** Logistic regression of ED variables for the diagnosis of COVID, excluding those defined only by clinical judgement. Odds ratio and confidence interval (CI at 95%) are shown and relative *p* values.

## Data Availability

Ethic committee does not allow sharing individual data. However, aggregated data and data dictionaries defining each field in the set will be made available on reasonable written request to the corresponding author.

## Abbreviations

SARS-CoV-2: Severe acute respiratory syndrome coronavirus 2
COVID-19: Coronavirus Disease 19
ED: Emergency Departments
RT-PCXR: Reverse Transcriptase Polymerase Chain Reaction
CT: Computed Tomography
GGO: Ground-Glass Opacity
CPAP: Continue Positive Airway Pressure
NIMV: Non-Invasive Mechanical Ventilation
IMV: Invasive Mechanical Ventilation
