# Supplementary material for: The broad spectrum of COVID-like patients initially negative at RT-PCR testing: a cohort study
Source: BMC Public Health. 2022 Jan 7;22:45. doi: 10.1186/s12889-021-12409-w (PMC8740875; doi:10.1186/s12889-021-12409-w)
Supplement: Supplementary file 1 — Additional file 1: Table S1. Sensitivity analyses. Data are described in the two subgroups COVID-19 (excluding those defined only by clinical judgement) and NOT-COVID defined at the end of the hospital stay by clinical judgment or further testing. Data are expressed as absolute frequencies and percentages (in brackets) for categorical variables and as medians and Interquartile Ranges [IQR]. Comparisons are made with: #: Wilcoxon Rank Sum Test; °: Chi-Square test; §: Fisher’s Exact test. Table S2. Logistic regression of ED variables for the diagnosis of COVID, excluding those defined only by clinical judgement. Odds ratio and confidence interval (CI at 95%) are shown and relative p values. [file 12889_2021_12409_MOESM1_ESM.docx]

Table S1 Sensitivity analyses. Data are described in the two subgroups COVID-19 (excluding those defined only by clinical judgement) and NOT-COVID defined at the end of the hospital stay by clinical judgment or further testing. Data are expressed as absolute frequencies and percentages (in brackets) for categorical variables and as medians and Interquartile Ranges [IQR]. Comparisons are made with : #: Wilcoxon Rank Sum Test; °: Chi-Square test; §: Fisher’s Exact test.

|  | **COVID-19** | **COVID-free/other than COVID** | **P** |
| --- | --- | --- | --- |
| **n** | 38 | 49 |  |
| **age (years)** | 60.54 [53.18, 80.33] | 78.09 [72.01, 84.33] | 0.002^#^ |
| **sex** |  |  | 0.313° |
| F | 13 ( 34.2) | 22 ( 44.9) |  |
| M | 25 ( 65.8) | 27 ( 55.1) |  |
| ***Criteria*** |  |  |  |
| **Epidemiological Criteria** |  |  | 0.310° |
| No | 22 ( 57.9) | 23 ( 46.9) |  |
| Yes | 16 ( 42.1) | 26 ( 53.1) |  |
| **Epidemiological Criteria Specification** |  |  | 0.051^§^ |
| Contact to COVID-19 | 7 ( 18.4) | 6 ( 12.2) |  |
| Nursing home resident | 4 ( 10.5) | 11 ( 22.4) |  |
| Repeated health care services users | 1 ( 2.6) | 8 ( 16.3) |  |
| Health worker | 4 ( 10.5) | 1 ( 2.0) |  |
| None | 22 ( 57.9) | 23 ( 46.9) |  |
| **Clinical criteria** |  |  | 0.071^§^ |
| No | 1 ( 2.6) | 8 ( 16.3) |  |
| Yes | 37 ( 97.4) | 41 ( 83.7) |  |
| ***Clinical criteria specification*** |  |  |  |
| **Cough, Dyspnoea** |  |  | 0.375° |
| No | 12 ( 31.6) | 20 ( 40.8) |  |
| Yes | 26 ( 68.4) | 29 ( 59.2) |  |
| **Fever** |  |  | 0.001° |
| No | 10 ( 26.3) | 31 ( 63.3) |  |
| Yes | 28 ( 73.7) | 18 ( 36.7) |  |
| **Hyposmia, Hypogeusia** |  |  | 0.314^§^ |
| No | 35 ( 92.1) | 48 ( 98.0) |  |
| Yes | 3 ( 7.9) | 1 ( 2.0) |  |
| **Nausea, Vomiting, Diarrhoea** |  |  | 1.000^§^ |
| No | 36 ( 94.7) | 46 ( 93.9) |  |
| Yes | 2 ( 5.3) | 3 ( 6.1) |  |
| **Respiratory failure** |  |  | 0.701° |
| No | 19 ( 50.0) | 26 ( 54.2) |  |
| Yes | 19 ( 50.0) | 22 ( 45.8) |  |
| **Number of symptoms** | 2.00 [2.00, 2.75] | 2.00 [1.00, 2.00] | 0.008^#^ |
| **Worsening of PO2/FiO2 without cause** |  |  | 0.561° |
| No | 24 ( 70.6) | 27 ( 64.3) |  |
| Yes | 10 ( 29.4) | 15 ( 35.7) |  |
| ***Comorbidities*** |  |  |  |
| **Hypertension** |  |  | 0.598° |
| No | 20 ( 52.6) | 23 ( 46.9) |  |
| Yes | 18 ( 47.4) | 26 ( 53.1) |  |
| **Cardiopathy** |  |  | 0.982° |
| No | 28 ( 73.7) | 36 ( 73.5) |  |
| Yes | 10 ( 26.3) | 13 ( 26.5) |  |
| **COPD** |  |  | 0.006° |
| No | 35 ( 92.1) | 33 ( 67.3) |  |
| Yes | 3 ( 7.9) | 16 ( 32.7) |  |
| **Lung fibrosis** |  |  | 0.381^§^ |
| No | 37 ( 97.4) | 45 ( 91.8) |  |
| Yes | 1 ( 2.6) | 4 ( 8.2) |  |
| **Lung Cancer** |  |  | 0.629^§^ |
| No | 37 ( 97.4) | 46 ( 93.9) |  |
| Yes | 1 ( 2.6) | 3 ( 6.1) |  |
| **Cancer** |  |  | 0.946° |
| No | 32 ( 84.2) | 41 ( 83.7) |  |
| Yes | 6 ( 15.8) | 8 ( 16.3) |  |
| **Immunodepression** |  |  | 1.000^§^ |
| No | 34 ( 89.5) | 43 ( 87.8) |  |
| Yes | 4 ( 10.5) | 6 ( 12.2) |  |
| **Neurological disorders** |  |  | 0.221° |
| No | 28 ( 73.7) | 30 ( 61.2) |  |
| Yes | 10 ( 26.3) | 19 ( 38.8) |  |
| **Diabetes or other metabolic conditions** |  |  | 0.706° |
| No | 24 ( 63.2) | 29 ( 59.2) |  |
| Yes | 14 ( 36.8) | 20 ( 40.8) |  |
| **Renal Failure** |  |  | 0.309^§^ |
| No | 34 ( 89.5) | 40 ( 81.6) |  |
| Yes | 4 ( 10.5) | 9 ( 18.4) |  |
| ***Laboratory Tests*** |  |  |  |
| **Total WBC count /µl** | 6.25 [4.71, 8.51] | 10.90 [7.48, 15.20] | <0.001^#^ |
| **Lymphocyte count/ µl** | 1135.00 [820.00, 1655.00] | 1410.00 [740.00, 1870.00] | 0.659^#^ |
| **LDH U/L** | 277.00 [222.00, 321.00] | 272.00 [217.25, 427.50] | 0.912^#^ |
| **PCR mg/dL** | 4.07 [1.61, 12.44] | 3.48 [0.49, 12.60] | 0.529^#^ |
| **PCT ng/mL** | 0.05 [0.03, 0.08] | 0.16 [0.03, 1.05] | 0.034^#^ |
| **PaO2/FiO2 at arrival** | 319 [276 - 374] | 322 [247 - 373] | 0.616^#^ |
| ***Diagnostic Tests*** |  |  |  |
| **Chest X ray (n=107)** |  |  | 0.556° |
| Pneumonia consolidation | 10 ( 27.0) | 9 ( 19.6) |  |
| Interstitial syndrome | 11 ( 29.7) | 10 ( 21.7) |  |
| Aspecific findings | 8 ( 21.6) | 15 ( 32.6) |  |
| Normal CXR | 8 ( 21.6) | 12 ( 26.1) |  |
| **Lung ultrasound (n=88)** |  |  | 0.099^§^ |
| Consolidation | 8 ( 24.2) | 5 ( 15.2) |  |
| Monolateral mild interstitial syndrome | 2 ( 6.1) | 6 ( 18.2) |  |
| Bilateral severe interstitial syndrome | 15 ( 45.5) | 7 ( 21.2) |  |
| Pleural effusion | 4 ( 12.1) | 6 ( 18.2) |  |
| Normal Lung Ultrasound | 4 ( 12.1) | 9 ( 27.3) |  |
| **CT scan (n=85)** |  |  | <0.001^§^ |
| Typical pattern | 21 ( 84.0) | 8 ( 22.9) |  |
| Atypical pattern | 1 ( 4.0) | 14 ( 40.0) |  |
| Undetermined | 3 ( 12.0) | 11 ( 31.4) |  |
| Normal CT scan | 0 ( 0.0) | 2 ( 5.7) |  |
| **Irregular pleural line (n=89)** |  |  | 0.114^§^ |
| No | 30 ( 88.2) | 33 (100.0) |  |
| Yes | 4 ( 11.8) | 0 ( 0.0) |  |
| ***Clinical course*** |  |  |  |
| **CPAP/NIV** |  |  | 0.798° |
| No | 31 ( 81.6) | 41 ( 83.7) |  |
| Yes | 7 ( 18.4) | 8 ( 16.3) |  |
| **CPAP/NIV duration days** | 6.00 [5.00, 10.50] | 5.50 [2.75, 8.50] | 0.449^§^ |
| **ED outcome** |  |  | 1.000^§^ |
| Discharged | 3 ( 7.9) | 3 ( 6.1) |  |
| Admitted | 35 ( 92.1) | 46 ( 93.9) |  |

Table S2 Logistic regression of ED variables for the diagnosis of COVID, excluding those defined only by clinical judgement. Odds ratio and confidence interval (CI at 95%) are shown and relative p values.

|  |  | **OR** | **95 % confidence interval** | **pvalue** |
| --- | --- | --- | --- | --- |
| **COPD** | *yes vs no* | 2.71 | (0.26-35.55) | 0.41 |
| **fever** | *yes vs no* | 7.76 | (1.04-108.15) | 0.07 |
| **Lung ultrasound** | *Consolidation vs Normal Lung Ultrasound* | 0.88 | (0.03-21.09) | 0.94 |
|  | *Monolateral mild interstitial syndrome vs Normal Lung Ultrasound* | 0.43 | (0.02-9.57) | 0.59 |
|  | *Bilateral severe interstitial syndrome vs Normal Lung Ultrasound* | 4.40 | (0.32-79.63) | 0.28 |
|  | *Pleural effusion vs Normal Lung Ultrasound* | 0.79 | (0.02-24.29) | 0.89 |
| **CT scan** | *Typical vs Other* | 12.16 | (1.65-140.96) | 0.02 |
| **WBC** |  | 0.87 | (0.68-1.04) | 0.18 |
| **age** |  | 1.00 | (0.93-1.08) | 0.98 |
| **sex** | *Male vs Female* | 2.80 | (0.29-41.45) | 0.40 |
